# Diet-induced increase in plasma oxidized LDL promotes early fibrosis in a renal porcine auto-transplantation model

**DOI:** 10.1186/1479-5876-12-76

**Published:** 2014-03-22

**Authors:** Nicolas Chatauret, Frédéric Favreau, Sebastien Giraud, Antoine Thierry, Ludivine Rossard, Sylvain Le Pape, Lilach O Lerman, Thierry Hauet

**Affiliations:** 1 INSERM, U1082, Ischémie-reperfusion en transplantation rénale, Université de Poitiers, Faculté de Médecine et de Pharmacie, Poitiers 86000, France; 2 CHU de Poitiers, Laboratoire de biochimie, Poitiers 86000, France; 3 Division of Nephrology and Hypertension, Mayo Clinic, Rochester, Minnesota 55905, USA; 4 INRA, UE1372 GenESI, Plateforme Ibisa, Surgères, France

**Keywords:** Hypercholesterolemia, Oxidized LDL, Ischemia reperfusion, Kidney transplantation, Preclinical model, Extracellular matrix

## Abstract

**Background:**

In kidney transplantation, the prevalence of hypercholesterolemia as a co-morbidity factor known to affect graft function, is rising due to the increased number of older donors in response to organ shortage as well as to the hyperlipidemic effects of immunosuppressors in recipient. This study aimed to characterize the effects of hypercholesterolemia on renal graft outcome, investigating the role of oxidized low-density lipoprotein (OxLDL).

**Methods:**

*In vivo,* we used a porcine preclinical model of renal auto-transplantation modulated by two experimental diets: a normal (n = 6) or a hyperlipidemic diet (n = 5) maintained during the 3 month follow-up after the surgical procedure. Kidney function and OxLDL levels were monitored as well as fibrosis, LOX-1 and TGF beta signaling pathways. *In vitro*, we used human artery endothelial cells subjected to OxLDL to investigate the TGF beta profibrotic pathway and the role of the scavenger receptor LOX-1.

**Results:**

Hyperlipidemic diet-induced increase in plasma OxLDL levels at the time of surgery correlated with an increase in proteinuria 3 months after transplantation, associated with an early graft fibrosis combined with an activation of renal TGF beta signaling. These data suggest a direct involvement of OxLDL in the hyperlipidemic diet-induced activation of the pro-fibrotic TGF beta pathway which seems to be activated by LOX-1 signaling. These results were supported by studies with endothelial cells incubated in culture medium containing OxLDL promoting TGF beta expression inhibited by LOX-1 antibody.

**Conclusions:**

These results implicate OxLDL in the hyperlipidemic diet-promoted fibrosis in transplanted kidneys, suggesting LOX-1 as a potential therapeutic target and reinforce the need to control cholesterol levels in kidney transplant recipients.

## Background

In kidney transplantation, the prevalence of hypercholesterolemia as a co-morbidity factor is rising due to the increased number of older donors in response to organ shortage as well as to the hyperlipidemic effects of immunosuppressors in recipient [1,2]. In general, hypercholesterolemia is associated with increased circulating levels of oxidized low density lipoproteins (OxLDL) [3]. These modified lipoproteins are involved in endothelial cell dysfunction [4], the first cell type subjected to ischemia and reperfusion injury (IRI) in organ transplantation.

In normocholesterolemic renal transplantation, endothelial activation is involved in inflammation and fibrosis development [5]. Despite the known deleterious effects of hypercholesterolemia on endothelial cell function and the importance of this cell type in IRI, few data are available on the consequences of hypercholesterolemia during organ transplantation [6]. In living-donor kidney transplantation, donor hypercholesterolemia is associated with a reduction in the 2-year renal graft function [7]. In deceased-donor transplantation, pre-transplant hypercholesterolemia in the recipient increases the rate of acute rejections [8] and the risk of late graft loss [9], whereas post-transplantation hypercholesterolemia is associated with chronic allograft dysfunction [10]. Bosmans et al. have previously suggested a role of OxLDL in poor renal allograft outcome in hyperlipidemic recipients [11] indicating the possible involvement of OxLDL in kidney IRI in hypercholesterolemic conditions.

In the kidney, hyperlipoproteinemia and their subsequent oxidation are associated with glomerular capillary dysfunction in rodents and severe glomerulosclerosis in dyslipidemic patients due to lipid deposits in glomeruli [12,13]. Experimental studies in pigs have demonstrated that diet-induced hypercholesterolemia led to renal endothelial dysfunction associated with vascular and microvascular remodeling, inflammation, and kidney fibrosis [14,15]. Thus, hypercholesterolemia can affect endothelial cell function and accelerate tissue remodeling in the kidney graft.

In atherosclerosis, the lectin-like OxLDL receptor-1 (LOX-1) plays a direct role in plaque formation [16]. This scavenger receptor is expressed on endothelial cells [17], smooth muscle cells and macrophages [18]. In these cells, binding of OxLDL to LOX-1 leads to reactive oxygen species (ROS) generation combined with NFκB activation [19] leading to oxidative stress and endothelial activation [20]. In cardiac fibroblasts, LOX-1 activation has been linked to collagen synthesis *via* an interaction between LOX-1-NADPH oxidase-TGFβ (Transforming Growth Factor beta) leading to an activation of the Mitogen-activated protein kinase (MAPK) pathway [21] establishing a possible link between the OxLDL signaling pathway and irreversible tissue fibrosis.

Although dyslipidemia is recognized as a non-immunologic factor negatively affecting early graft function [8], the consequences on renal graft outcome remain to be clarified. In this study, we hypothesized that a high-fat diet (HD), started before transplantation and maintained after surgery, increases circulating levels of OxLDL, affects endothelial cell functions, and irremediably accelerates interstitial fibrosis development in auto-transplanted porcine kidneys.

## Methods

### Animal model and surgical procedures

Male Large White pigs were fed a standard (ND) or a high-fat diet (HD, standard diet + 20% Lard and 2% cholesterol) immediately after weaning and maintained until euthanasia [14]. The renal auto-transplantation model was performed when the animals reached 37-46 kg (3 months old) as previously described in accordance with the guidelines of the French Ministries of Agriculture and Research, and the institutional committee for the use and care of laboratory animals (CEEA Poitou-Charentes, project reference number: CE2012-4) [5,22,23]. Briefly, the left kidney was removed, flushed with 300 ml of UW preservation solution and preserved at 4°C in the same solution in static conditions for 24 hours. On the day of transplantation, the right kidney was removed and the left kidney grafted mimicking the nephron mass in the transplanted situation. Two experimental groups were studied: ND + Tx: transplanted kidneys removed 3 months after surgery from animals fed a standard diet (n = 6), HD + Tx: transplanted kidneys removed 3 months after surgery from animals fed a high-fat diet (n = 5). One transplanted HD pig died before completion of the study due to surgical complications and was not included in data analysis. Plasma creatinine, cholesterol and urinary proteins were measured using an automatic analyzer (Modular, Roche Diagnostic, France). OxLDL (Diasorin, Antony, France) and superoxide dismutase (SOD) activity (Cayman, Montigny Le Bretonneux, France) were measured in plasma.

### Immunohistopathological studies

Paraffin-embedded sections (3 μm) of renal cortical samples were examined under blinded conditions by a pathologist and a nephrologist. As described previously, the level of tubulo-interstitial fibrosis were investigated using Sirius red staining [24] and tissue remodeling by immunohistochemical assessment of vimentin expression (1/500, Cell Marque, Rocklin, CA, USA).

Frozen cortex sections (5 μm) were used to investigate LOX-1 and TGFβ expression by double immunofluorescence localization. We used a rabbit primary antibody at 1/100 (Abcam, Paris, France) and a goat anti-rabbit secondary antibody coupled to Alexa 488 fluorochrome (1/1000, Life Technologies, Saint Aubin, France) for LOX-1 expression and a mouse primary antibody at 1/100 (Santa Cruz, CA, USA) and a goat anti-mouse secondary antibody coupled to Alexa 568 Fluorochrome (1/1000, Life Technologies) for TGFβ.

### Western blotting procedure

A standard Western blotting protocol was used as described previously [5,25] with antibodies against TGFβ (1:600), matrix metalloproteinase 2 (MMP2, 1:200) (Santa Cruz, CA, USA); connective tissue growth factor (CTGF, 1:500) (Biovision, Mountain View, CA, USA), LOX-1 (1:1000) (R&D System), bone morphogenetic protein-7 (BMP-7, 1:5000) (AbDSerotec, Minneapolis, MN, USA), nuclear factor-kappa B (NFκB, 1:1000), its inhibitor kappa B alpha (IκBα, 1:200), Phospho-P38 (1:1000) (Millipore, Billerica, MA, USA), NADP(H) oxidase subunit Gp91phox (1:500, BD Transduction Laboratories, France). Loading controls were β actin (1:3000, Sigma Aldrich, France) or P38 (1:1000, Millipore). Appropriate HRP-coupled secondary antibodies (1:5000 to 1:10 000, GE Healthcare, France) were used to detect the band by chemiluminescence with ECL plus (GE Healthcare, France). Intensities of the protein bands were determined and quantified using AlphaEase FC software (Alpha Innotech Corporation, San Leandro, CA).

### Human LDL purification and oxidation

Human LDL were isolated by sequential ultracentrifugation and oxidized by UV-C irradiation as previously described [26]. LDL oxidation level was verified by quantification of the thiobarbituric-acid reacting substances (TBARS) [27]. This oxidation protocol led to an average TBARS concentration of 14.28 ± 2.21 μM.

### In vitro incubation of OxLDL on human aortic endothelial cells: effect of LOX-1 antibody

Human aortic endothelial cells (HAEC), obtained from Gibco (France), were cultured with M200 medium (Gibco) supplemented with 10% fetal bovine serum (Invitrogen, France) in a humidified atmosphere at 5% CO_2_ and 37°C. The cells were used for the experiments after 4 to 5 passages. For the time course of 24 h, OxLDL’s effects on LOX-1 and TGF β protein expressions were evaluated in HAECs treated with culture medium supplemented or not with OxLDL (25 μg/mL) [28,29]. We also evaluated TGFβ secretion in culture medium with a Duoset Elisa kit from R&D System (France). The effect of LOX-1 antibody (R&D System), selected for its ability to block receptor-ligand interaction, was tested in these different experimental conditions.

### Statistical methods

Results are shown as mean ± SEM. We used a student t-test for two-group comparisons or a Mann–Whitney test when the variance was not equal between the groups. Correlation studies were performed using linear regression. All statistical analyses were performed using the NCSS Software (Version 07.1.20, Kaysville, UT, USA). Statistical significance was accepted for *p* < 0.05.

## Results

### High-fat diet promoted increases in plasma OxLDL levels, worsened proteinuria but did not affect creatinine excretion recovery after kidney transplantation

**Figure 1:**
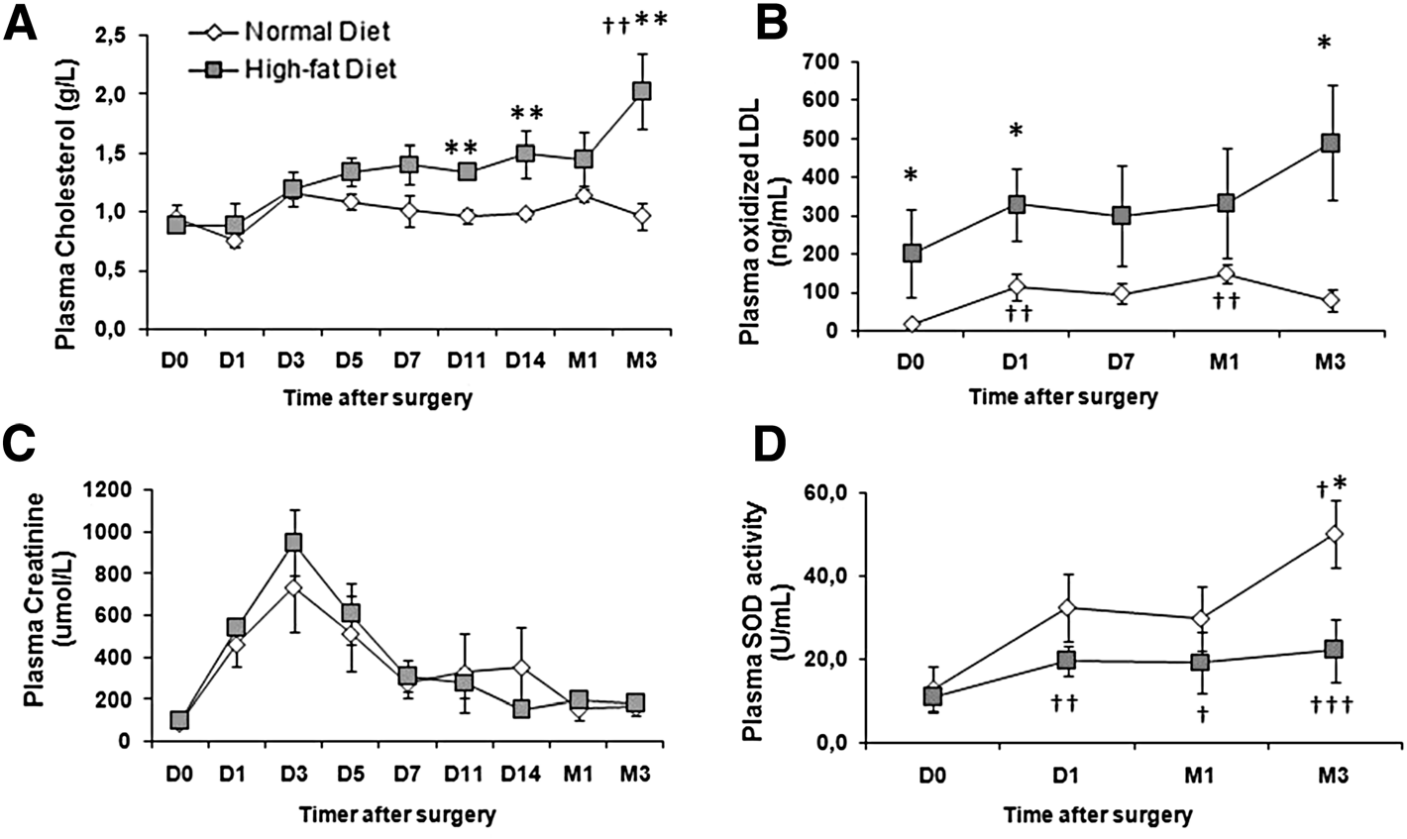
**High-fat diet increased plasma cholesterol and OxLDL levels in transplanted pigs. A)** Time course evolution of plasmatic total cholesterol levels measured on the day of transplantation (D0) and after surgery, in pigs fed either a normal (white) or a high-fat diet (grey); **B)** Time course evolution of plasma OxLDL levels. High-fat diet did not change graft function recovery assessed by plasma creatinine level monitoring, **C)** Time course evolution of plasmatic creatinine measured on the day of transplantation (D0) and after surgery; **D)** Time course determination of plasma SOD activity. Values significantly different from the normal diet group are indicated by *: p < 0.05; **: p < 0.01 and from the D0 time point by †: p < 0.05; ††: p < 0.01; †††: p < 0.001.

**Figure 2:**
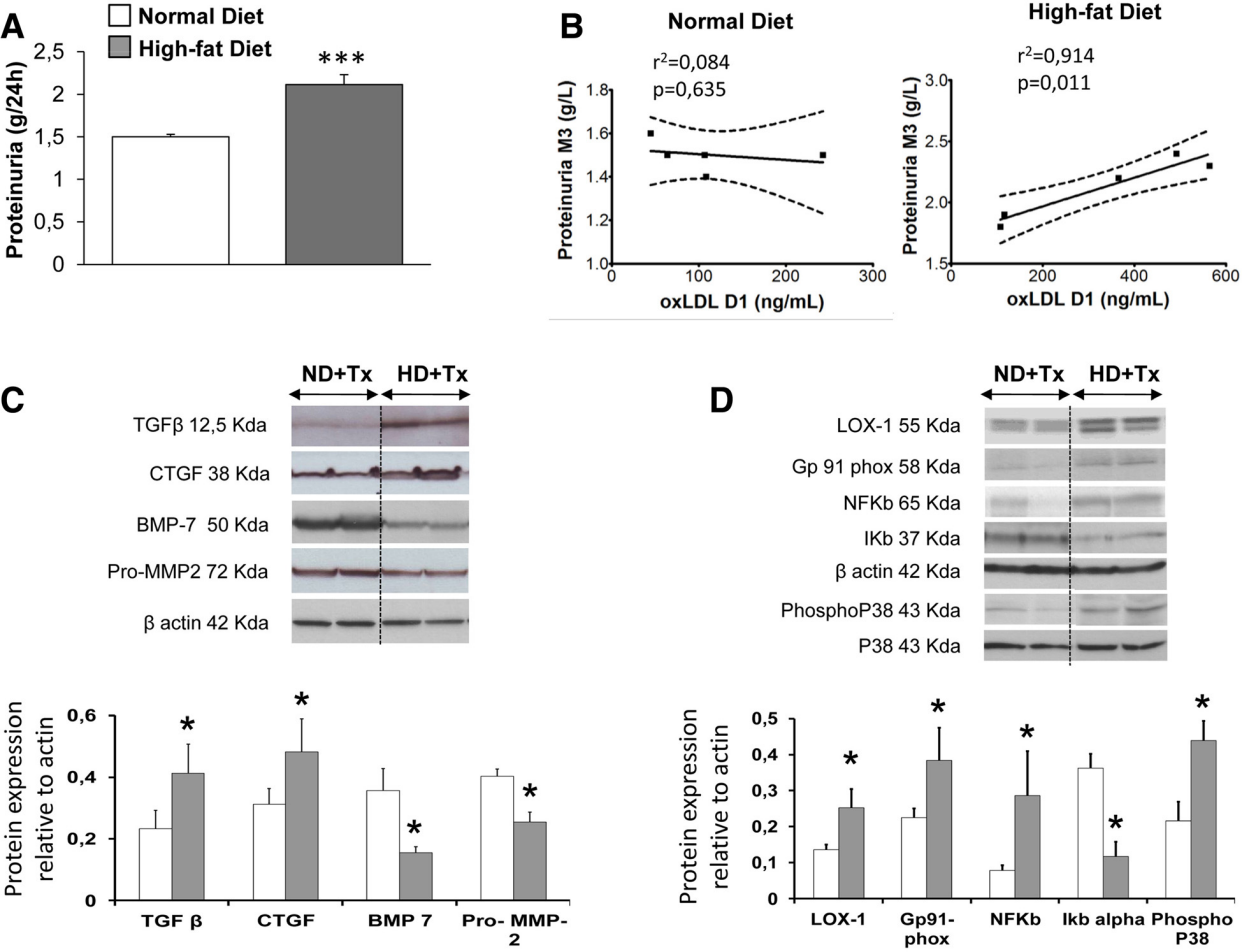
**High-fat diet was associated with a greater proteinuria and with LOX-1 and TGFβ pathways activation in renal cortex 3 months after transplantation. A)** Proteinuria levels in the two experimental groups 3 months after surgery; Values significantly different from the normal diet group are indicated by ***: p < 0.001; **B)** Linear regression of the plasmatic OxLDL levels measured at D1 and the proteinuria determined at M3 in the normal diet group (Left panel) and the high-fat diet group (Right Panel). The straight line represents the regression curve and dotted lines represent the 95% confidence interval; **C)** Immunoblots and densitometric quantification of TGFβ signaling (TGFβ, CTGF, BMP-7) and matrix homeostasis (pro-Matrix Metalloproteinase 2, pro-MMP2); and **D)** LOX-1 intracellular signaling (Gp91phox NADPH oxidase subunit, NFκB-IκBα, PhosphoP38) in renal cortex. Values significantly different from the normal diet group are represented by *: p < 0.05.

Although swine body weights were not significantly different between the groups on the day of surgery, HD animals exhibited a higher body weight at the end of the experiments (Table 1, p < 0.05). After renal transplantation, plasma cholesterol levels were significantly increased in the HD + Tx group after the first week following transplantation (p < 0.05) and were further elevated 3 months post-transplantation (Figure 1A). In the HD + Tx animals plasma levels of OxLDL remained statistically more elevated at day 1 and 3 months after surgery in comparison with the ND + Tx animals (p < 0.05, Figure 1B). This enhanced oxidative stress in the HD + Tx group was supported by a concomitant decrease in plasma SOD activity by 3 months (p < 0.05, Figure 1D, Table 1). In terms of kidney function, although serum creatinine increased after transplantation, there was no statistical difference among groups in its peak levels or recovery profile (Figure 1C). Similarly, diuresis recovery was not different between the two groups (Table 1). HD pigs exhibited elevated proteinuria 3 months after reperfusion compared to ND pigs (p < 0.05, Figure 2A). Linear regression analysis revealed a significant correlation between the circulating levels of OxLDL measured at day 1 post-surgery and the proteinuria determined at 3 months (r^2^ = 0.91, p = 0.01) in the HD group only (Figure 2B). These results suggest that OxLDL may be linked to renal graft survival and function.

**Table 1:** Kidney function and blood metabolites of transplanted animals fed a normal or a high-fat diet

	**Day 0**		**M3**	
	**Normal diet**	**High-fat diet**	**Normal diet**	**High-fat diet**
Body weight (Kg)	42.3 ± 1.2	40.9 ± 0.9	109.3 ± 3.8	129.0 ± 2.9*
Diuresis (ml/24 h)	2867 ± 145	2567 ± 88	3416 ± 487	2166 ± 364
Creatininemia, (μmol/L)	95 ± 2	99 ± 5	197 ± 22	184 ± 6
Plasma SOD activity (U/mL)	10.3 ± 3.9	10.9 ± 3.0	37.9 ± 7.4	22.2 ± 7.7*

*p < 0.05 vs Normal diet.

### HD concomitantly activated LOX-1 and TGFβ pathways

As mentioned above, HD animals presented, during the post-surgical follow-up, elevated plasma OxLDL levels in comparison to ND animals (p < 0.05, Figure 1B). This pro-oxidative milieu was accompanied in kidney graft, 3 months after surgery, by significant increases in pro-fibrotic TGFβ (p < 0.05) and its downstream effector CTGF (p < 0.05) protein levels and decreases in protein levels of the anti-fibrotic factor BMP-7 (2-fold, p < 0.05, Figure 2C). In addition, reduced Pro-MMP2 protein levels (an enzyme degrading the extracellular matrix) (p < 0.05) were observed in HD + Tx in comparison to ND + Tx animals (Figure 2C). The increase in circulating OxLDL levels in the HD + Tx group was accompanied by 2 fold- and 1.5- fold-upregulations of LOX-1 and Gp91phox protein levels respectively (p < 0.05 vs ND + Tx, Figure 2D). This was associated with significant increases in the expression of LOX-1 downstream effectors including NFκB and P38 proteins (p < 0.05) and a 4-fold decrease in the expression of the NFκB inhibitor Iκb-α (p < 0.05, Figure 2D), all of which have been implicated in the LOX-1 signaling pathway [21]. These observations suggest that a hyperlipidemic diet under our experimental conditions induced concomitant increases in oxidative stress and pro-fibrotic pathway signaling potentially stimulating fibrosis development after transplantation.

### HD promoted kidney graft fibrosis and tissue remodeling

**Figure 3:**
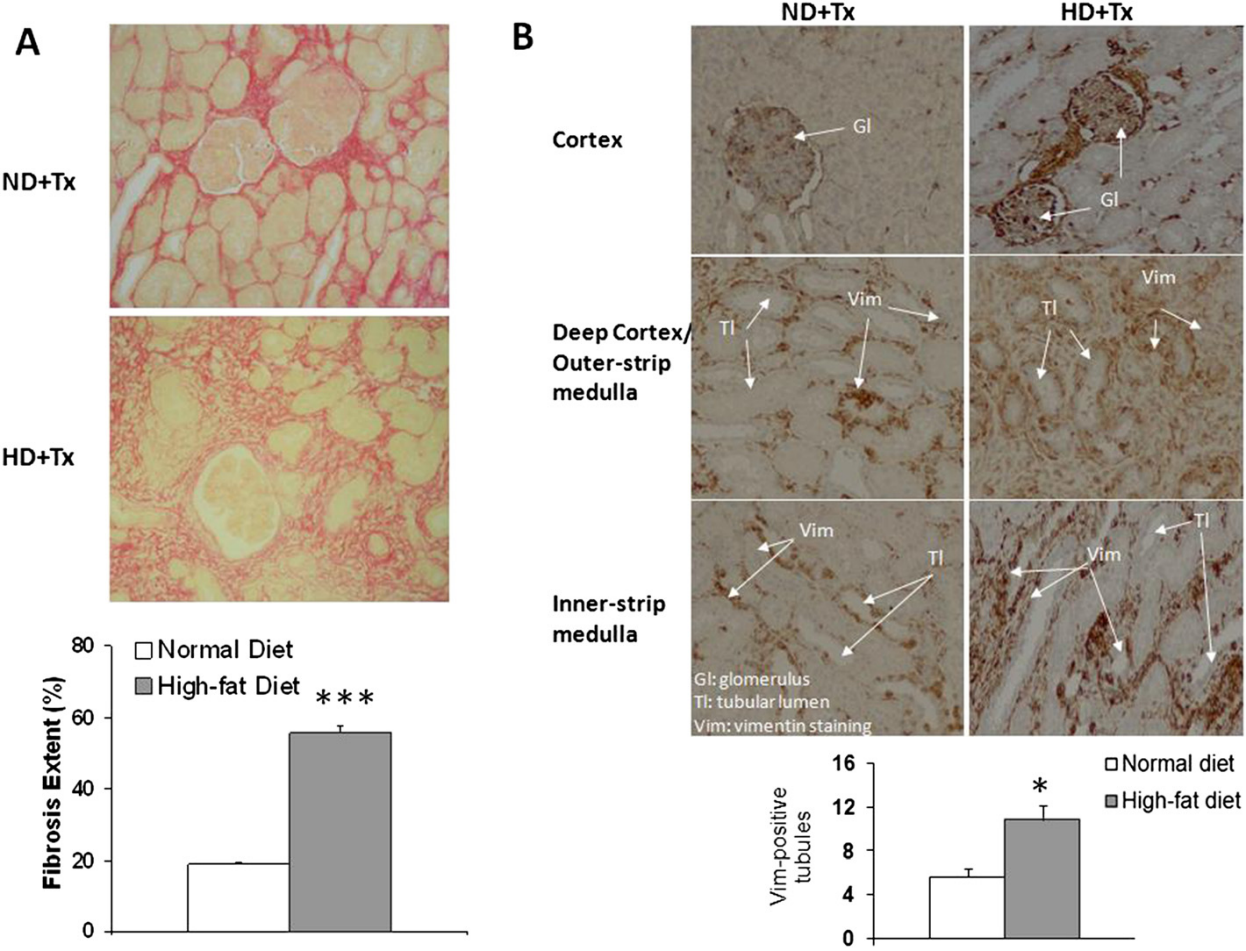
**High-fat diet increased renal interstitial fibrosis extent and the number of vimentin positive tubules.** Representative pictures (200X) of typical Sirius red staining **(A)** in transplanted pigs fed a normal or a high-fat diet. Histogram represents the percentage of fibrosis extent. Values significantly different from the normal diet group are represented by ***: p < 0.001. **B)** Representative pictures (200X) of vimentin staining revealed by diaminobenzidinine immunohistochemistry in transplanted pigs fed a normal or a high-fat diet. Arrows indicate glomerulus (Gl), tubules (Tl) and vimentin (Vim) localization. Histogram represents the quantification of the number of vimentin-positive tubules. Values significantly different from the normal diet group are represented by *: p < 0.05.

**Figure 4:**
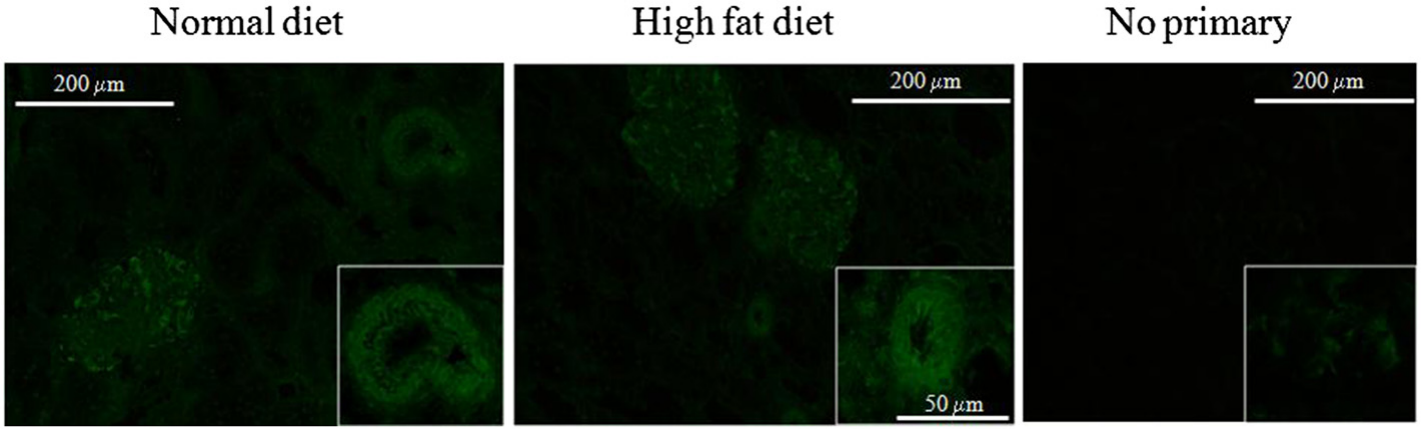
**Immunofluorescence localization of LOX-1 in the endothelium of intrarenal arteries.** Representative pictures of immunofluorescence localization of LOX-1 in kidney cortical samples 3 months after transplantation in animals fed either a normal or a high-fat diet.

**Figure 5:**
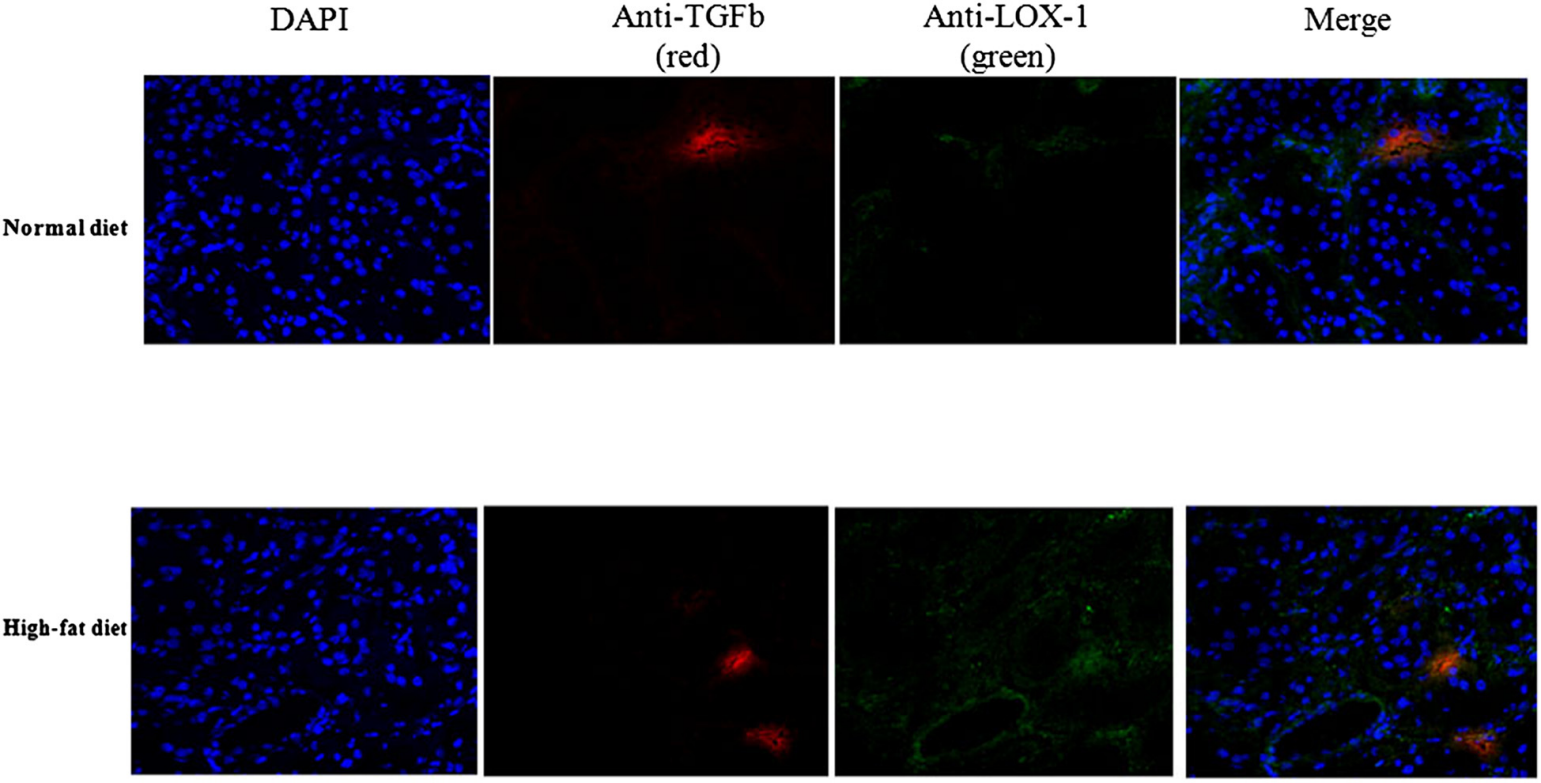
**Confocal analysis of TGFβ and LOX-1 expressions.** Representative pictures of double immunofluorescence localization of LOX-1(green) and TGFβ (red) in kidney cortical samples 3 months after transplantation in animals fed either a normal or a high-fat diet (Magnification x20). Colocalization of TGFβ and LOX-1 expressions (yellow) was observed around peritubular capillaries by confocal microscopy. Nuclei were stained with DAPI (Blue).

Three months after transplantation, red Sirius staining showed that HD + Tx cortex presented a 3-fold increase in fibrosis in comparison with the ND + Tx animals (p < 0.001, Figure 3A). Furthermore, HD + Tx pigs exhibited a higher number of vimentin-positive tubules supporting tissue remodeling (Figure 3B). Immunofluorescence experiments revealed that LOX-1 was localized mainly on vascular structures and glomeruli in the kidneys independently of the diet 3 months after transplantation (Figure 4). We also observed, by confocal analysis a co-localization of LOX-1 and TGFβ around peritubular capillaries 3 months after surgery independently to the diet (Figure 5). These data support the stipulation that HD promotes graft fibrosis and suggests interplay between OxLDL-LOX-1 and TGFβ.

### LOX-1-blocking antibody inhibited the OxLDL-induced TGFβ secretion in HAEC

**Figure 6:**
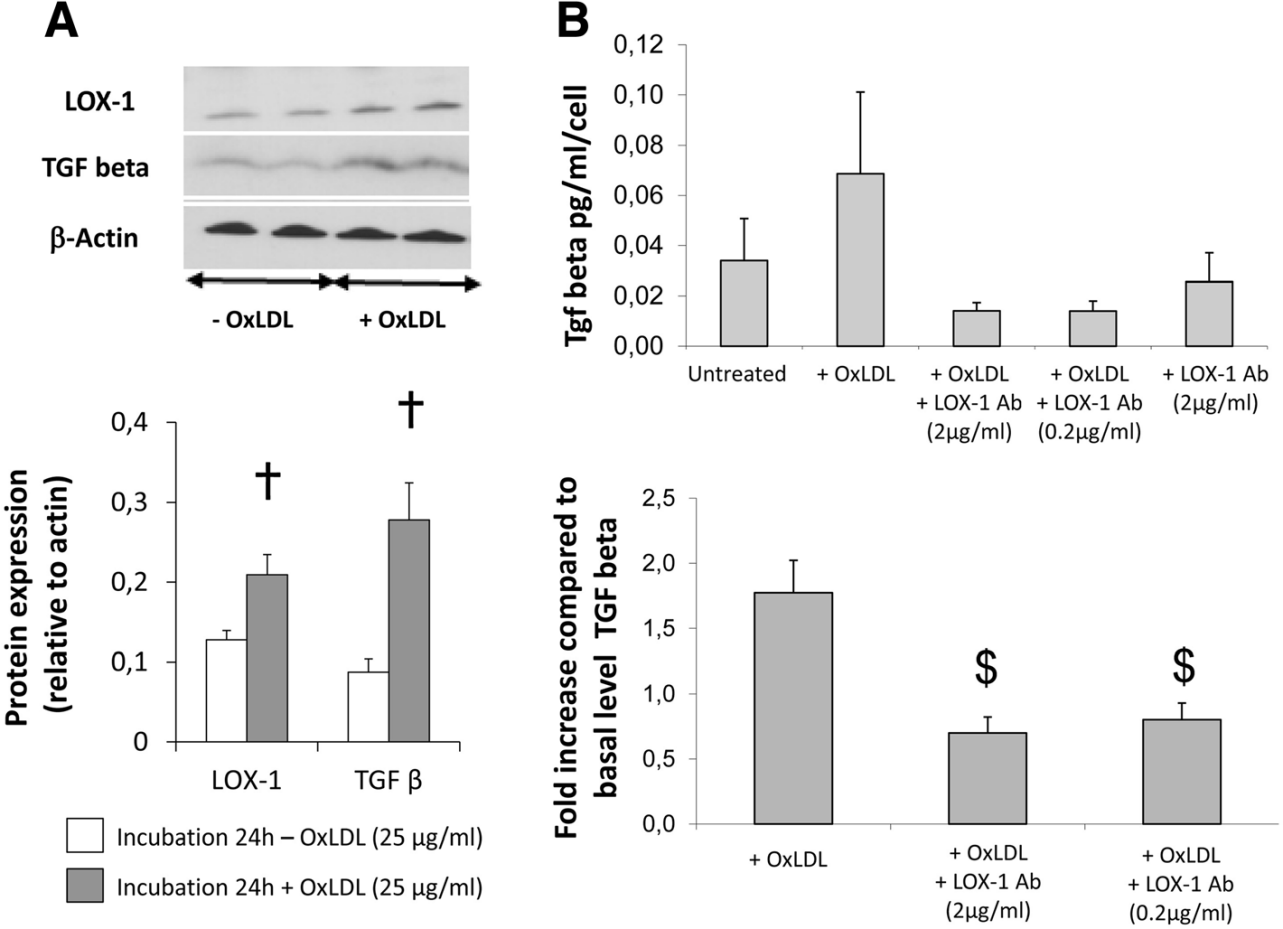
**LOX-1 antibody blocked TGFβ secretion induced by OxLDL in human artery endothelial cells. A)** Effects of OxLDL incubation during 24 h in endothelial cells on LOX-1 and TGFβ protein expressions, and **B)** on TGFβ secretion in culture medium supplemented with OxLDL with or without neutralizing antibody against LOX-1 (results obtained from two independent experiments). Values significantly different from the culture medium without OxLDL are indicated by †: p < 0.05; and from the culture medium without LOX-1 antibody by $: p < 0.05.

To evaluate the direct role of OxLDL in the HD-induced TGFβ overexpression observed *in vivo* in fibrotic kidney graft, we treated HAECs with OxLDL. OxLDL treatment led to respectively 1.6- and 3-fold increases in LOX-1 and TGFβ protein levels in comparison to PBS treated cells (p < 0.05, Figure 6A). This was associated with a 1.8-fold increase in TGFβ levels in the culture medium (Figure 6B). Addition of LOX-1-blocking antibody in the medium prior to OxLDL treatment prevented the OxLDL-mediated induction of TGFβ secretion (Figure 6B). This abolition of OxLDL-induced TGFβ secretion *in vitro* suggests a direct effect of OxLDL on TGFβ production *via* LOX-1, offering a plausible hypothesis to explain the increase in cortical TGFβ levels observed in transplanted animals fed a hyperlipidemic diet.

## Discussion

We demonstrated that diet-induced hypercholesterolemia was associated with significant increases in circulating levels of OxLDL. These HD-induced increases were not associated with alterations in kidney function recovery after transplantation but led to a 2.5-fold increase in the interstitial fibrosis extent and enhanced proteinuria 3 months after surgery in the hypercholesterolemic animals. This increased fibrosis extent, in the HD-animals, was linked to concomitant activations of TGFβ and LOX-1 signaling pathways, suggesting a probable association of the OxLDL-LOX-1 in tissue fibrosis development. Immunohistochemical studies revealed that LOX-1 expression was mainly found in the vascular compartment, including the endothelium involving endothelial cells in the HD effect. The hypothesis of a direct involvement of the OxLDL-LOX1 signaling pathway in the activation of the TGFβ signaling pathway in the pro-fibrotic kidney graft is suggested by *in vitro* results demonstrating that blocking LOX-1 prevented the OxLDL-induced increase in TGFβ secretion by arterial endothelial cells. Taken together, the increased fibrosis extent and over activation of TGF-β signaling pathway in hypercholesterolemic conditions suggest a poor long-term graft outcome in the HD animals.

Hypercholesterolemia increases LDL susceptibility to oxidation [30] and therefore production of plasmatic OxLDL [31,32]. Diet-induced increases in circulating levels of OxLDL have been reported in pigs in the past [33]. In this study, we hypothesized that OxLDL could directly exacerbate fibrosis injuries in kidney graft. Transplanted kidney was exposed to fibrosis tissue spread which promotes a hypoxic milieu due to capillary rarefaction as well as alterations in oxygen diffusion capacity which is an additional cause of fibrosis. To assess this hypothesis, we compared *in vitro* the effects of OxLDL exposure on endothelial cells. OxLDL exposure induced TGF-β protein expression and enhanced endothelial LOX-1 expression.

*In vivo,* we investigated in pigs, fed with either a normal or a high-fat diet, the levels of plasma OxLDL during the first three months of graft follow-up. After transplantation in normocholesterolemic conditions, pigs exhibited a significant increase in plasmatic OxLDL levels at day 1 and 30 as previously reported for kidney IR in rodents [34] or suggested in human kidney transplantation by the presence of elevated levels OxLDL auto-antibodies [35]. These increases in plasma OxLDL levels are in accordance with the well-characterized oxidative stress induced by the ischemia-reperfusion sequence in normocholesterolemic conditions [36]. In hypercholesterolemic animals, plasma OxLDL and SOD levels were further elevated and decreased respectively during the 3 months post-surgery indicating a greater oxidative stress in these animals. Oxidative stress is one of the major deleterious mechanisms involved in IRI and delayed graft function [37-39]. In addition, HD did not significantly alter kidney function recovery evaluated by creatininemia or diuresis during the 3-month follow-up period despite a greater graft fibrosis in comparison to ND animals. This absence of correlation between early graft function and fibrosis extent occurs also in the clinic [40]. Prevalence of interstitial fibrosis and tubular atrophy has been reported to be 25% at 3 months and 50% at 2 years in 41 patients with normal graft function [41]. Nonetheless, graft fibrosis has been reported to reduce long-term graft survival [40]. The 3-month follow-up in the present work is likely too short to observe an impact of HD on basal kidney function.

Interestingly in the HD group, circulating levels of OxLDL, evaluated one day after transplant surgery, were significantly correlated with the proteinuria present 3 months later, suggesting a detrimental role of OxLDL on graft outcome. Plasma OxLDL levels may be a relevant parameter to monitor just after transplantation in the recipient to predict graft outcome. Also, this suggests that therapeutic interventions aimed at reducing the levels of these modified lipoproteins in the recipient should be started as early as possible. Fibrosis is considered to be the major process leading to renal graft loss. The involvement of TGFβ and its signaling pathway in the etiology of kidney graft fibrosis is well characterized [42-44]. In the present study, the HD-associated increase in fibrosis may be linked to the elevated levels of plasma OxLDL. Indeed, Hu et al. have established a link between LOX-1-NADPH oxidase and the TGFβ-mediated collagen synthesis in cardiac fibroblasts [21]. *In vivo,* the direct involvement of LOX-1 in IRI and remodeling has been previously reported in normocholesterolemic mouse hearts [45,46]. In the present work, HD + Tx induced concomitant increases in LOX-1 and TGFβ signaling pathways represented by NFκB, phospho P38 and the gp91phox NAD(P)H oxidase subunit up-regulations and decreased IκB expression for the LOX-1 signaling pathway and by TGFβ and CTGF up-regulations as well as BMP-7 and Pro-MMP2 down-regulations for the TGFβ signaling pathway indicating a reduced capacity for matrix degradation giving rise to a pro-fibrotic milieu. TGFβ is a pivotal growth factor involved in several processes linked to IRI and fibrosis. The parallel increases in collagen and vimentin expressions in our study may partially be explained by TGFβ involvement in vimentin expression, a mesenchymal cell marker, indicating tissue remodeling and dedifferentiation of tubular cells towards mesenchymal cell types leading to fibrosis [47]. These observations suggest a poor outcome of kidney graft in high-OxLDL conditions.

**Figure 7:**
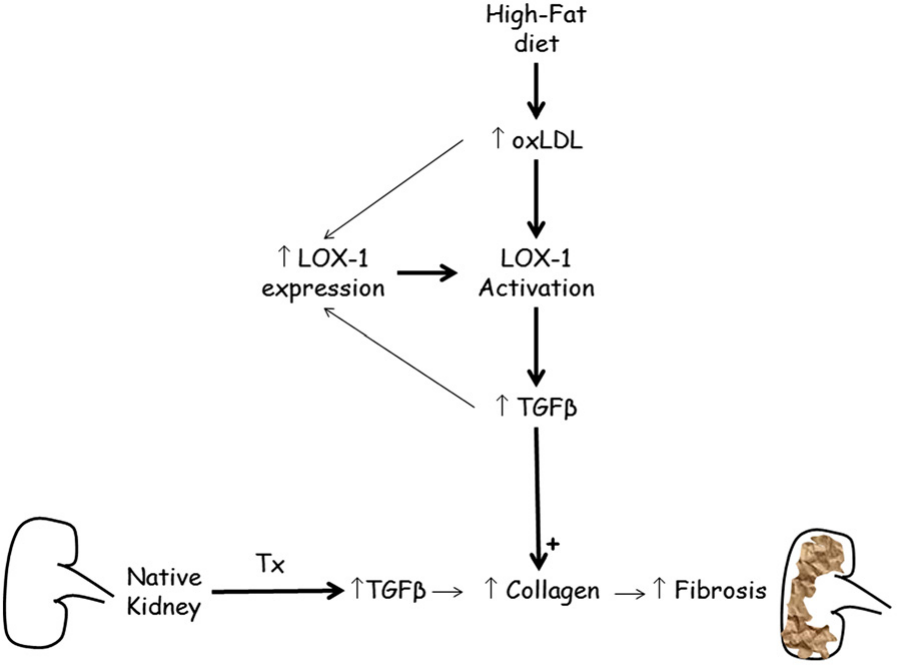
**Proposed mechanism of diet-induced fibrosis in transplanted kidneys.** In normocholesterolemic condition, transplantation leads to an increase in TGFβ levels resulting in an increase in vimentin positive tubules and collagen production which are both involved in fibrosis development. Data from this study indicate that the increase in plasma OxLDL levels induced by high-fat diet leads to LOX-1 pathway activation by ligand fixation and promotes an increase in LOX-1 protein content *via* either Ox-LDL alone or TGFβ stimulation in artery endothelial cells, which in turn over-activates the TGFβ signaling pathway. This activation acts in synergy with transplantation to increase fibrosis development.

These data suggest that an association between OxLDL, LOX-1 and TGFβ is present in HD-grafted kidneys. LOX-1 can be viewed as a mediator of endothelial dysfunction [48]. Immunofluorescent staining in transplanted kidneys revealed an intense expression of LOX-1 in the endothelium of intrarenal arteries as previously shown in hyperlipidemic pig kidneys [49-51]. These results were supported by colocalization of TGFβ and LOX-1 expressions around peritubular capillaries. To characterize the role of OxLDL in fibrosis development observed *in vivo,* we investigated the direct involvement of LOX-1 in the TGFβ pathway in arterial endothelial cells : the first target of ischemia reperfusion injury in solid organ transplantation. A culture medium supplemented with OxLDL induced, in endothelial cells, concomitant overexpressions of TGFβ and LOX-1 proteins levels. Both OxLDL and TGFβ have been shown to induce LOX-1 expression [21,52-54] and in this case increased LOX-1 expression could be mediated either directly by Ox-LDL or indirectly *via* an Ox-LDL-induced increase in TGFβ. Nonetheless, blocking human LOX-1 with an antibody prior to OxLDL addition prevented the increase in TGFβ secretion in the culture medium supporting the stipulation that induction of TGFβ expression was the consequence of LOX-1 activation by the Ox-LDL in this *in vitro* setting. The proposed mechanism of diet-induced fibrosis in transplanted kidneys is summarized in Figure 7. Briefly, in normocholesterolemic conditions, the transplantation process leads to an increase in TGFβ levels resulting in an increase in vimentin positive tubules and collagen production which are both involved in fibrosis development as previously described in our model [55]. In case of a high-fat diet, the increase in plasma OxLDL levels leads to LOX-1 pathway activation by ligand fixation and promotes increase in LOX-1 protein content via either Ox-LDL alone or TGFβ stimulation in artery endothelial cells which in turn over-activates the TGFβ signaling pathway. This activation acts in synergy with the transplantation process to increase fibrosis. This proposed mechanism involving a direct relationship between LOX-1 and fibrosis development is supported by recent reports indicating that LOX-1 abrogation reduced tissue remodeling in mice heart [56] and kidneys [57].

Our study was limited by the use of young pigs with a short term high-fat diet started before surgery, and by the relatively short duration of the post-transplant follow-up in comparison to the human disease. In addition, human renal transplantation is not only associated with dyslipidemia but also with immunosuppressor therapy or other concurrent or pre-existing pathophysiological conditions such as hypertension or diabetes which impair the renal microvasculature and likely modulate its response to transplantation. Nevertheless, the renal structure and function in the swine model are similar to human kidneys [58], and our results bear relevance and may shed light on the short-term negative impact of diet-induced increase in OxLDL circulating levels on renal IRI following transplantation. In addition, our model is characterized by a relatively short-term exposure to hypercholesterolemia and by the absence of chronic vascular injury. To our knowledge, this study is the first to report, in a large animal model, a link between hypercholesterolemia and fibrosis development in kidney transplantation involving OxLDL and the LOX-1 receptor, highlighting a pathophysiological mechanism starting at an early stage, in the absence of chronic injury and without detectable change on the monitoring of the renal function. In humans, the benefits of cholesterol lowering therapy have been investigated in a randomized control trial [59]. This study revealed that treatment of renal graft recipient with fluvastatin, starting 5 years after transplantation, did not improve graft function or graft loss even though there was a significant reduction in the risk of cardiac death [60]. Taking into account the early changes supported by this study in pigs, the fluvastatin treatment in this clinical trial may need to be initiated earlier to prevent the deleterious consequences of hypercholesterolemia. These observations strongly suggest that cholesterol lowering- or LOX-1 blocking therapies should be initiated as early as possible in kidney graft recipients. This study supports the assessment of these therapeutic strategies in humans or in large animal models. Such preclinical models are of interest because they allow a rapid transfer for clinical application. Complementary studies are warranted to focus on the effect of HD in donors and consequences in recipient.

## Conclusion

The significant correlation between plasma OxLDL and proteinuria observed in the present work, as well as the concomitant activation of LOX-1 and TGFβ signaling pathways *in vivo* and the direct interaction between LOX-1 and TGFβ secretion *in vitro*, implicate OxLDL in the HD-induced fibrosis and tissue remodeling observed as early as 3 months after renal transplantation. Taken together, these results suggest that diet could affect graft outcome, identify LOX-1 as a therapeutic target of interest and emphasize the need to better control either cholesterol or OxLDL plasma levels in recipient with dietetic and therapeutic measures at the early stage of renal transplantation.

## Abbreviations

OxLDL: Oxidized low-density lipoprotein; IRI: Ischemia and reperfusion injury; LOX-1: lectin-like OxLDL receptor-1; ROS: Reactive oxygen species; TGFβ: Transforming growth factor beta; MAPK: Mitogen-activated protein kinase; HD: High-fat diet; ND: Standard diet; SOD: Superoxide dismutase; MMP2: Matrix metalloproteinase 2; CTGF: Connective tissue growth factor; BMP-7: Bone morphogenetic protein-7; NFκB: Nuclear factor-kappa B; IκBα: Kappa B alpha inhibitor; TBARS: Thiobarbituric-acid reacting substances.

## Competing interests

The authors declare that they have no competing interests.

## Authors’ contributions

NC carried out protein, histochemical and immunohistochemical studies, performed the statistical analysis, and drafting the manuscript. FF was involved in conception and design of this study, acquisition of data, analysis and interpretation, and also in drafting the manuscript, GS has been involved in revising the manuscript critically for important intellectual content, AT has been involved in drafting the manuscript, RL carried out protein studies. SL carried out cell culture, analysis and interpretation of results, LOL has been involved in revising the manuscript critically for important intellectual content, TH has involved in conception and design of this study, acquisition of data, analysis and interpretation, and also in drafting and revising the manuscript. All authors read and approved the final manuscript.
